# Characterization of Chemically Induced Liver Injuries Using Gene Co-Expression Modules

**DOI:** 10.1371/journal.pone.0107230

**Published:** 2014-09-16

**Authors:** Gregory J. Tawa, Mohamed Diwan M. AbdulHameed, Xueping Yu, Kamal Kumar, Danielle L. Ippolito, John A. Lewis, Jonathan D. Stallings, Anders Wallqvist

**Affiliations:** 1 Department of Defense Biotechnology High Performance Computing Software Applications Institute, Telemedicine and Advanced Technology Research Center, U.S. Army Medical Research and Materiel Command, Fort Detrick, Maryland, United States of America; 2 U.S. Army Center for Environmental Health Research, Fort Detrick, Maryland, United States of America; University of Navarra School of Medicine and Center for Applied Medical Research (CIMA), Spain

## Abstract

Liver injuries due to ingestion or exposure to chemicals and industrial toxicants pose a serious health risk that may be hard to assess due to a lack of non-invasive diagnostic tests. Mapping chemical injuries to organ-specific damage and clinical outcomes via biomarkers or biomarker panels will provide the foundation for highly specific and robust diagnostic tests. Here, we have used DrugMatrix, a toxicogenomics database containing organ-specific gene expression data matched to dose-dependent chemical exposures and adverse clinical pathology assessments in Sprague Dawley rats, to identify groups of co-expressed genes (modules) specific to injury endpoints in the liver. We identified 78 such gene co-expression modules associated with 25 diverse injury endpoints categorized from clinical pathology, organ weight changes, and histopathology. Using gene expression data associated with an injury condition, we showed that these modules exhibited different patterns of activation characteristic of each injury. We further showed that specific module genes mapped to 1) known biochemical pathways associated with liver injuries and *2*) clinically used diagnostic tests for liver fibrosis. As such, the gene modules have characteristics of both generalized and specific toxic response pathways. Using these results, we proposed three gene signature sets characteristic of liver fibrosis, steatosis, and general liver injury based on genes from the co-expression modules. Out of all 92 identified genes, 18 (20%) genes have well-documented relationships with liver disease, whereas the rest are novel and have not previously been associated with liver disease. In conclusion, identifying gene co-expression modules associated with chemically induced liver injuries aids in generating testable hypotheses and has the potential to identify putative biomarkers of adverse health effects.

## Introduction

Exposure to toxic chemicals is a major environmental health hazard for military personnel, potentially causing both acute and long-term adverse health effects [1]. Accurately diagnosing exposure injuries through non-invasive tests would allow for early intervention, treatment, damage assessment, and prediction of potential for recovery [2], [3]. There are multiple theoretical and practical challenges in deriving serum or urine biomarkers that could address these issues [2], [3]. Here, we are primarily addressing issues related to identifying sets of genes that are characteristic of and specific to chemical exposure conditions and liver-injury outcomes.

A toxic insult triggers numerous interconnected biochemical signaling and response pathways at the cellular, organ, and systemic levels. Xenobiotic metabolism, damage control and repair, and inflammation are all central ways for the biological system to cope with chemical stress. Part of this response is encoded and executed through transcriptional control, and a multitude of studies has used gene expression microarrays to characterize this toxicogenomic response [4]–[9]. The concept of a finite set of molecular toxicity pathways that govern these stress-responses has been used as an argument for using cell-based systems to understand and identify chemical toxicity. However, cell-based assays and studies often fail to mimic all of the effects of toxicants at the organ or whole-body level. Here, we have examined a large toxicogenomics data collection, DrugMatrix [10], hosted by the National Institute of Environmental Health Sciences (NIEHS) in an effort to conceptually connect molecular toxicity pathways to co-expressed gene modules and link these pathways to specific injuries. The DrugMatrix database contains normalized, organ-specific data on chemically induced gene expression changes and associated changes in clinical pathology, organ weight, and histopathology endpoints in male Sprague Dawley rats [10].

Co-expressed gene modules have been used to identify (classify) genes specific to tumors of certain cancers [11], as well as for repurposing drugs as cancer therapeutics [12]. Modules are typically constructed to represent injury or injured states based on activation or repression of the genes in the module. The key feature of a module is that the constituent genes share an expression pattern across a set of stress conditions. Conceptually, the simplest module could simply be the top differentially expressed genes under conditions causing injury. Computational methods that have been developed to create these modules, including hierarchical clustering [13], bi-clustering [14], [15], overlay of transcriptomics data to biological networks to create network modules [16], and use of gene signatures from classification models [17], such as support vector machines (SVMs) [18].

The conceptualization of molecular toxicity pathways allows us to define the desirable properties of gene modules in the context of biomarker discovery. Foremost, genes within a module should exhibit a high level of intra-module gene correlation, which implies (but does not guarantee) an underlying common regulatory biological process that governs their expression pattern. The correlated gene set in a module may not span all conditions in the study (not all toxicants induce the same response) and genes in one module may appear in other modules (genes may be part of multiple response pathways). An additional desirable module property is that gene regulation within the module is specific to the injury, e.g., regardless of which chemicals cause fibrosis in the liver, the gene module is activated in a similar manner, and, hence, is specific to fibrosis.

Given the role of the liver in detoxification and as a primary site of chemical injuries, we performed a bioinformatics analysis of all liver arrays run on the Affymetrix platform and their coupled clinical chemistry endpoints in DrugMatrix. We evaluated several methods for gene module construction in terms of injury specificity and intra-module gene correlation. Of the methods tested, we found that the iterative signature algorithm (ISA) [14], [15] maximized these parameters and we used it to compute 78 gene co-expression modules associated with the liver data in DrugMatrix. Each of these modules was then associated with a specific set of activation patterns for 25 diverse injury endpoints (indicators) categorized from clinical pathology, organ weight changes, and liver histopathology [19].

We found that the activation patterns of the modules were characteristic for each injury indicator. Furthermore, when we mapped module genes to biochemical pathways, we found that different injuries could be characterized not only by a difference in co-regulation module activation patterns, but also by their different utilization of these biochemical pathways. These biochemical pathway associations with injuries are well documented in the literature, and many of the specific module gene sets have curated relationships with liver disease in the Comparative Toxicogenomics Database [20]. Hence, the modules we constructed retained part of the broadly underlying disease biology and a response context consistent with the notion of molecular pathways of toxicity in the liver. Based on this rationale, we examined the potential for deriving biomarker hypotheses based on the constructed modules to create signature gene sets for liver fibrosis, steatosis, and general liver injury. The bulk of the selected genes (58 out of 69) had no known associations with liver disease; therefore, they provide important avenues of future validation and biomarker discovery.

In conclusion, gene co-expression modules can be used to characterize chemically induced liver injuries and provide a rational basis for selecting putative biomarkers, a necessary step in the development of diagnostic tests for monitoring adverse health effects due to environmental toxicant exposures.

## Materials and Methods

### Data

We used data from DrugMatrix [21], a public available database that contains matched data associating chemical exposures with *1*) transcriptomic changes in multiple tissues/organs of male Sprague Dawley rats and *2*) clinical pathology, histopathology, and organ weight assessments. The specimens used to generate the database were collected at multiple time points after administration of drugs and toxicants at different concentrations and from multiple organs such as liver, kidney, heart, bone marrow, spleen, thigh muscle, blood, and brain. Microarray gene expression experiments were then executed in triplicates for selected tissue samples and clinical endpoints were measured, although not for all possible drug-exposure conditions in all organs. Based on the Natsoulis *et al*. [22] analysis, we focused on a data-rich set of 2,218 Affymetrix microarrays from DrugMatrix run on liver tissue. The data span 25 general and liver-specific toxicity endpoints and nine structure-activity sets derived from well-defined chemical drug and toxicant classes. This data set contained 200 different and diverse chemicals. **Table 1** shows these clinical endpoints designated as general clinical pathology, body organ weight, and liver histopathology. Note that the category *Eosinophilia* is listed under histopathology as it was categorized from the histopathology inspection, i.e., hepatocellular eosinophilia. **Table 2** lists the drug-activity classes and the drugs/toxicants used to define these sets.

**Table 1:** DrugMatrix [21], [22] clinical injury indicators.

Injury indicators
**General clinical pathology (CP)**
1. Corpuscular hemoglobin decrease
2. Corpuscular hemoglobin concentration decrease
3. Corpuscular hemoglobin concentration decrease, days 5/7
4. Basophil increase
5. Lipase increase
6. Lymphocyte decrease
7. Glucose increase
8. Leukocyte increase
9. Albumin increase
10. Creatinine increase
11. Glucose decrease
12. Monocyte increase
13. Total protein increase
14. Hemoglobin decrease
15. Leukocyte count decrease
16. Alkaline phosphatase decrease
**Body organ weight (OW)**
1. Liver weight decrease
2. Liver weight increase
3. Spleen weight decrease
**Liver histopathology (LH)**
1. Periportal lipid accumulation
2. Eosinophilia
3. Centrilobular inflammatory cell infiltrate
4. Periportal fibrosis
5. Centrilobular lipid accumulation
6. Periportal hypertrophy

**Table 2:** DrugMatrix [21], [22] structure-activity classified drugs and toxicants.

Stressors	Exemplar chemicals
1. Estrogen receptor agonists	Estriol, beta-estradiol, ethinylestradiol, mestranol
2. GR-MR agonists	Betamethasone, cortisone, dexamethasone, fluocinolone acetonide, hydrocortisone
3. PDE4 inhibitors	Piclamilast, roflumilast, rolipram
4. HMG-CoA reductase inhibitors	Cerivastatin, fluvastatin
5. DNA alkylators	Aflatoxin B1, 2-acetylaminofluorene, hydrazine, 4,4'-methylenedianiline, n-nitrosodiethylamine
6. PPAR alpha agonists or fibric acid	Bezafibrate, cofibric acid, gemfibrozil, nafenopin, pirinixic acid
7. Toxicant heavy metals, all doses	Lead(IV) acetate, sodium arsenite
8. Toxicant heavy metals, low dose	Lead(IV) acetate, sodium arsenite
9. H+/K+-ATPase inhibitors	Pentoprazole, rabeprazole

Each microarray corresponds to gene transcription changes in the liver as caused by a specific exposure scenario or “condition” versus control samples. Here, we defined “condition” as a specific organ-chemical-concentration-time combination. Following the nomenclature of Natsoulis *et al.* [22], injury indicators take on a value of +1 if a positive injury (abnormal) indication is recorded for that specific condition.

### Data processing

We downloaded the 2,218 liver microarray datasets run on Affymetrix GeneChip Rat Genome 230 2.0 Array from DrugMatrix [23]. We used the *ArrayQualityMetrics* [24] BioConductor package to assess the quality of the Robust Multi-Array Averaged (RMA) [25] pre-processed data. In this process, we found and removed 155 outlier arrays and renormalized the remaining data. After array-level filtering and normalization, we performed gene level filtering using the BioConductor package *genefilter* [26]. Specifically, we removed genes without Entrez IDs or with low variance across conditions. We implemented the low variance criteria from Bourgon *et al.* [27] by computing and sorting the expression variance of each gene over the complete condition set and removing the bottom half as low-variance genes. Additional filtering was performed using the default settings for the *affy* package from BioConductor to remove probe sets below a signal-to-noise threshold. The number of replicates for each condition that had a “Present” call was determined for each probe set. Only probe sets for which at least 25% of the conditions had “Present” calls for all replicates within a condition were retained for further analysis. In the rest of the paper, we have used the terms *gene id* and *probeset* interchangeably. When we discuss the gene expression or log ratio values, we refer only to probesets.

With the remaining genes and conditions, we calculated log ratios (LRs) for each gene as the difference between treatment and control RMA expression levels. We computed log_2_ expression values for treatment and control as averages over replicates. We assembled a log ratio matrix LR with rows defined by genes, columns defined by conditions, and the matrix elements, LR*_i,j_*_,_ defined as log ratios for genes *i* under conditions *j*. As a last step, we transformed the log ratios into Z-scores. The Z-score of gene *i* under condition *j* is given by
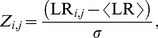
(1)where the average <…> runs over all genes *i* and conditions *j* in the data set, and *σ* denotes the standard deviation of the LR average. The resultant log-ratio Z-score matrix contained 7,826 genes by 640 conditions and the entire data set is provided in the Supporting Information as **Table S1**.

### Gene set selection procedures

We used six different methods to construct gene sets based on hierarchical clustering, protein-protein interaction (PPI) data, existing gene sets derived from the examined data, randomized data, highest fold-change selection, and the ISA. The latter algorithm partially uses the other gene sets as input for a more comprehensive gene set refinement.

#### Hierarchical clustering

We used the R package *Hclust* [13] to cluster the gene dimension of the log-ratio matrix. Each gene in this matrix was represented by a vector of 640 log_2_ ratio values, each value representing the response of the gene to the imposed condition (chemical, concentration, time, tissue). Using these vectors, we computed all gene-pair Pearson correlation coefficients. We used 1 minus the Pearson correlation (1 – *r*) as a distance metric between the genes, and we used average linkage to compute the distance between gene clusters. We utilized the *cutreeDynamic* function within the *dynamicTreeCut* [28] R package to automate extraction of clusters. The dynamic tree cut algorithm uses the cluster dendrogram to identify and split clusters into sub-clusters until the minimum cluster size threshold is reached. When implementing *cuttreeDynamic* we used the minimum cluster size set to *16*, *method* set to *hybrid*, *deepsplit* set to *True*, and the maximum cluster size set to *100*.

#### Protein-protein interaction (PPI) network gene sets

We mapped the Affymetrix Rat Genome 230 2.0 Array probe IDs to their human orthologs using the National Center for Biotechnology Information HomoloGene database (http://www.ncbi.nlm.nih.gov/homologene). A high-confidence human PPI network [29] was used to construct protein interaction gene sets. We defined a gene set as an individual protein and all of its directly interacting partners within the PPI network. We constructed 11,789 PPI-based gene sets in this way. We chose DrugMatrix microarrays associated with positive instances of the injury indicators, and we mapped these to the PPI gene sets. To score the 11,789 gene sets we determined the number of up- and down-regulated genes (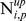 and 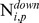) in each set *i* for a given injury indicator *p*. We converted these to Z-scores using the following equations: 
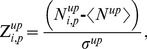
(2)

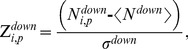
(3)where the average <…> and standard deviation σ were computed over 10^6^ permutations of the positive conditions associated with injury indicator *p*. To establish a reliable significance threshold for these scores, we ran the randomization experiment 100 times. Each time we determined the most significant positive Z-score and the most significant negative Z-score (using the randomized Z-score values) to form two groups with 100 Z-scores each. We sorted the up-regulated group in decreasing order and the down-regulated group in increasing order. Identification of the fifth entry in each list, i.e., the fifth percentile out of the 100 scores, allowed us to define a gene set Z-score threshold that produced an estimated maximum false positive rate of 5%.

#### Support vector machine (SVM) gene sets

We used the 34 signatures developed by Natsoulis *et al.*, [22] for predicting 25 injury endpoints, as well as the activity of nine selected chemical structure activity classes. These genes sets were developed for endpoint classifications using the DrugMatrix data, but, as shown by the authors, they also contain biological information relevant to the effects of the chemical.

#### Random (RAND) and maximum average Z (MAZ) gene sets

To generate random gene sets we used the *generate.seeds* routine, which is part of the *eisa* [15], [30] BioConductor package, to construct randomly selected sets of 100 genes.

To generate maximum average Z clusters (MAZ), we selected the positive class conditions associated with each injury indicator and sorted the genes in decreasing order by their average magnitude Z-scores across the condition set. We chose the top genes in the sorted list to generate the MAZ gene clusters.

#### Gene set refinement using the iterative signature algorithm (ISA) [14]

We used the R package *eisa* to generate ISA co-expression modules associated with the entire Z-score matrix of 7,826 genes by 640 conditions. We first ran *ISAIterate*, which requires a starter gene set *G_starter_* that is typically built using previous biological knowledge associated with the genes, e.g., using gene sets *g* from hierarchical clustering or KEGG pathway genes. An individual starter gene set was built using *N_starter_* genes and was defined as



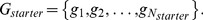
(4)


Each condition *c* was given a score *s^c^* using the average Z-score value of the starter genes for that condition:



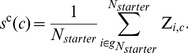
(5)


The conditions are relevant if their scores *s^c^(c)* were greater than *t_c_* standard deviations away from the mean score across all conditions. We denoted the set of *N_r_* relevant conditions as *C_relevant_*, which is formally written as



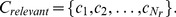
(6)


In the current work, we defined a relevant condition as one for which *t_c_* was equal to or greater than 1.8.

Each gene *i* was then scored as the weighted average of its Z-score values across the relevant conditions:



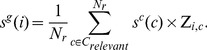
(7)


The genes were relevant if their scores *s^g^(i)* were more than *t_g_* standard deviations away from the mean score for that condition set. We identified the set of *N_g_* relevant genes as *G_relevant_* by setting *t_g_* to 3.5. The process was then iterated by substituting the starter gene set *G_starter_* with *G_relevant_* and recalculating all scores. The iterations were continued until the set of relevant genes *G_relevant_* and relevant conditions *C_relevant_* did not change by more than 1% in a given iteration.

A single starter gene set will converge to a particular co-expression module defined as a set of genes *G_relevant_* and associated set of conditions *C_relevant_* for which the gene expression values were correlated. Many starter gene sets and iterations were required to generate gene sets that can characterize all genes and conditions that the DrugMatrix data encompass. In order to avoid the creation of redundant modules, we pruned our results using the routine *ISAUnique*, with the parameter *cor.limit* set to its default value. To ensure that the gene sets were robust, i.e., the core module composition did not change when adding random genes, we used the routine *ISAFilterRobust* with default parameters.

As mentioned above, we used values of *t_g_* and *t_c_* set to 3.5 and 1.8, respectively. We determined these values after many trials of the ISA using fixed starter gene sets derived from HC, PPI networks, and SVMs in order to ensure that the modules were no larger than the size of an average KEGG pathway. At the same time, we maximized the module parameters for indicator specificity and intra-module gene correlation as is discussed below. **Script S1** in the Supporting Information provides the R script and the input files used for the generation of ISA modules.

### Module evaluation parameters

#### Specificity

The activation 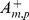 of module *m* associated with positive instances of injury indicator *p* is the average Z-score for all genes in the module *m* across all conditions with a positive instance of the injury and is given by



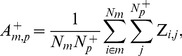
(8)where *N_m_* is the number of genes associated with module *m*, 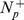 is the number of positive class conditions for indicator *p*, and the Z-score matrix elements are defined by Equation (1).

We assessed the statistical significance of the activation scores by calculating the distribution of all 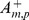 activation scores for all *m* and *p* pairs. The distribution of scores indicated that an absolute activation score of 1.5 or larger was associated with the ∼5%-tails of the near-normal distribution. We used activation scores larger than 1.5 in this work as indicative of a significant association between a module *m* and an injury indicator *p*.

The absolute value of the difference in the activation of module *m* between positive class instances of injury indicators *p* and *q* is



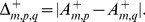
(9)


This was used to compute the specificity of module *m* to injury indicator *p*,



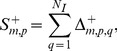
(10)where *N_I_* denotes the 25 injury indicators shown in **Table 1**. The maximum specificity to injury indicator *p* is
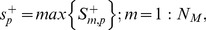
(11)where *N_M_* denotes the total number of modules and the global specificity is given by
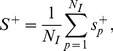
(12)with larger values of 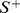 indicating module sets with higher injury-indicator specificity.

#### Intra-module gene correlation

The average Pearson correlation 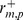 of genes in module *m* under conditions that cause positive instances of injury indicator *p* is,



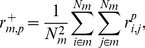
(13) where *m* and *p* are module and injury indicator indices, *N_m_* is the number of genes in module *m*, *i* and *j* are gene indices, and 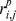 is the Pearson correlation between genes *i* and *j* across conditions that cause positive instances of injury indicator *p*.

The maximum intra-module gene correlation for injury indicator *p* is,



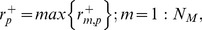
(14)where *N_M_* denotes the total number of modules and the global intra-module gene correlation R^+^ is,
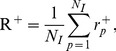
(15)where *N_I_* denotes the 25 injury indicators shown in **Table 1**. Larger values of 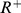 indicate module sets with higher intra-module gene correlation.

### Pathway association of gene co-expression modules

We mapped the genes in the co-expression modules to KEGG [31] pathways. We used Fisher's exact test with Bonferroni-corrected *p*-values to determine the statistical significance of the resulting pathways. We filtered the pathways using the following constraints: *1*) pathways must be associated with absolute module activation scores 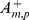 (Equation (8)) that are larger than 1.5 for conditions causing a particular injury type, *2*) Bonferroni-corrected *p*-values of the pathways must be smaller than 0.05, and *3*) pathways must contain at least six genes from a module to be mapped to it.

### Activation of individual genes under different injury conditions

Similar to the module activation defined in Equation (8), we can define the activation of a particular gene in response to an injury indicator. Thus, the activation 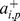 of gene *i* associated with positive instances of injury indicator *p* is given by



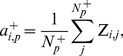
(16)where the summation runs over all 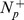 members of positive class conditions for indicator *p*, and the Z-score matrix elements are defined by Equation (1).

### Center gene selection

In order to simplify the selection of a gene from a module as a specific biomarker for a particular injury indicator, we introduced the concept of a representative *center* gene. For a given module *m* containing *N_m_* genes, we computed the Pearson correlation sum 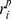 for each gene *i* and for a given injury indicator *p* as,
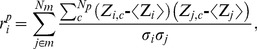
(17)where *i* and *j* denote gene indices, *N_p_* is the number of conditions that invoke an abnormal instance of injury indicator *p*, and the average <Z*_i/j_*> and standard deviations σ*_i/j_* were computed across the second index of the Z-score matrix as defined in Equation (1). Using the values of 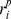 we determined the center gene of module *m* as the gene with the largest Pearson correlation sum. If the average Z-score of the center gene across conditions causing abnormal instances of the injury indicator was less than the module average, the gene was thrown out, and a new center gene was chosen as the gene with the next largest Pearson correlation sum with average activation larger than the module mean.

### External validation

We further evaluated the relevance of the periportal fibrosis and general liver injury gene signatures using external datasets collected from the Toxicogenomics Project-Genome Assisted Toxicity Evaluation System (TG-GATEs) database [32] and the Gene Expression Omnibus (GEO) [33]. All the external datasets utilized Affymetrix GeneChip Rat Genome 230 2.0 Arrays. For all the external datasets, we collected the raw CEL files and processed them in the same manner as described above for the DrugMatrix data.

The TG-GATEs database contains gene expression data from both *in vitro* and *in vivo* studies. It contains expression data from Sprague-Dawley rats and hepatocytes that have been exposed to 150 selected chemicals at different dose and time points. This database includes biochemistry and histopathology data associated with each exposure. We selected the exposures with a high dose (15 mg/kg) of naphthyl isothiocyanate at four, eight, and 15 days as an external validation set for our periportal fibrosis gene signature since these exposures produced observable periportal liver fibrosis. Next, we evaluated the periportal fibrosis gene signature in the GEO dataset - GSE13747 [34]. In this dataset, liver fibrosis was induced by bile duct ligation, and there were six replicates of liver fibrosis samples and six controls. For the genes in the periportal fibrosis gene signature, we compared their fold-change in the DrugMatrix study to the fold-change in these external sets using a Pearson correlation.

We used the GEO dataset - GSE5509 to validate the general liver injury gene signature [35]. In this dataset, rats were treated with three toxic compounds (α-naphthyl-isothiocyanate, dimethyl nitrosamine, and N-methyl formamide) and three non-toxic compounds (rosiglitazone, caerulin, and di-nitrophenol). There were five replicates of each chemical exposure. We evaluated the ability of the general liver injury gene signature to separate toxic and non-toxic compounds in this dataset. We used the classical multi-dimensional scaling (MDS) function in R to create the MDS plot.

## Results and Discussion

### Gene sets for module construction

The constructed Z-score matrix elements represent normalized gene activation patterns in liver tissues in response to different chemical exposure conditions. The matrix contained 7,826 genes arrayed in 640 different conditions and constitutes the coupled transcriptional response for multiple overlapping and intertwined toxic response mechanisms. As outlined in the Methods, we used multiple methods to construct co-expression gene sets that can represent these responses. Using hierarchical clustering, we generated 231 gene sets that each contained an average of 33 genes. The gene set construction method based on PPIs gave a total of 595 significantly up- or down-regulated gene sets with an average size of 50 genes. The previously constructed 34 gene sets from Natsoulis *et al.* [22] contained an average of 79 genes. Furthermore, we generated 34 maximum expression change gene sets each containing 50 genes and 100 random gene sets containing 100 genes each. We used the randomly constructed gene sets to assess the ability of the deployed methods to create co-expression modules above the random noise level (the null-hypothesis).

**Figure 1:**
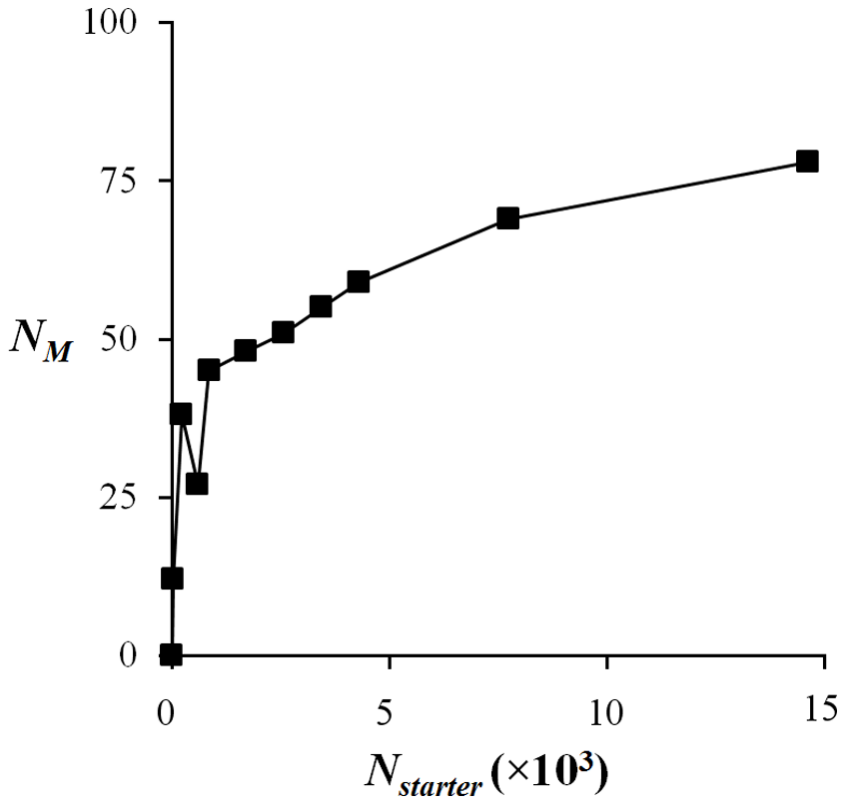
Iterative signature algorithm (ISA) module generation analysis. Number of iterative signature algorithm (ISA) modules *N_M_* as a function of the number of starter gene sets, *N_starter_*.

For the ISA gene set construction we used the entire Z-score matrix as input and defined the 859 initial starter gene sets using the hierarchical gene set clusters (231), protein-interaction derived gene sets (595), and the SVM gene sets from Natsoulis *et al.* [22]. We repeatedly expanded this set by adding 100 randomly selected genes to each starter gene set to generate thousands of starter gene sets, each derived from the original 860 gene sets. **Figure 1** shows the number of gene co-expression modules generated by ISA as a function of the number of starter gene sets input to ISA. At over 14,000 starter gene sets, we generated 78 co-expression modules with average size of 31 genes. Further expansion did not significantly increase the number of unique gene sets.

### Gene set evaluation using specificity and correlation metrics

**Figure 2:**
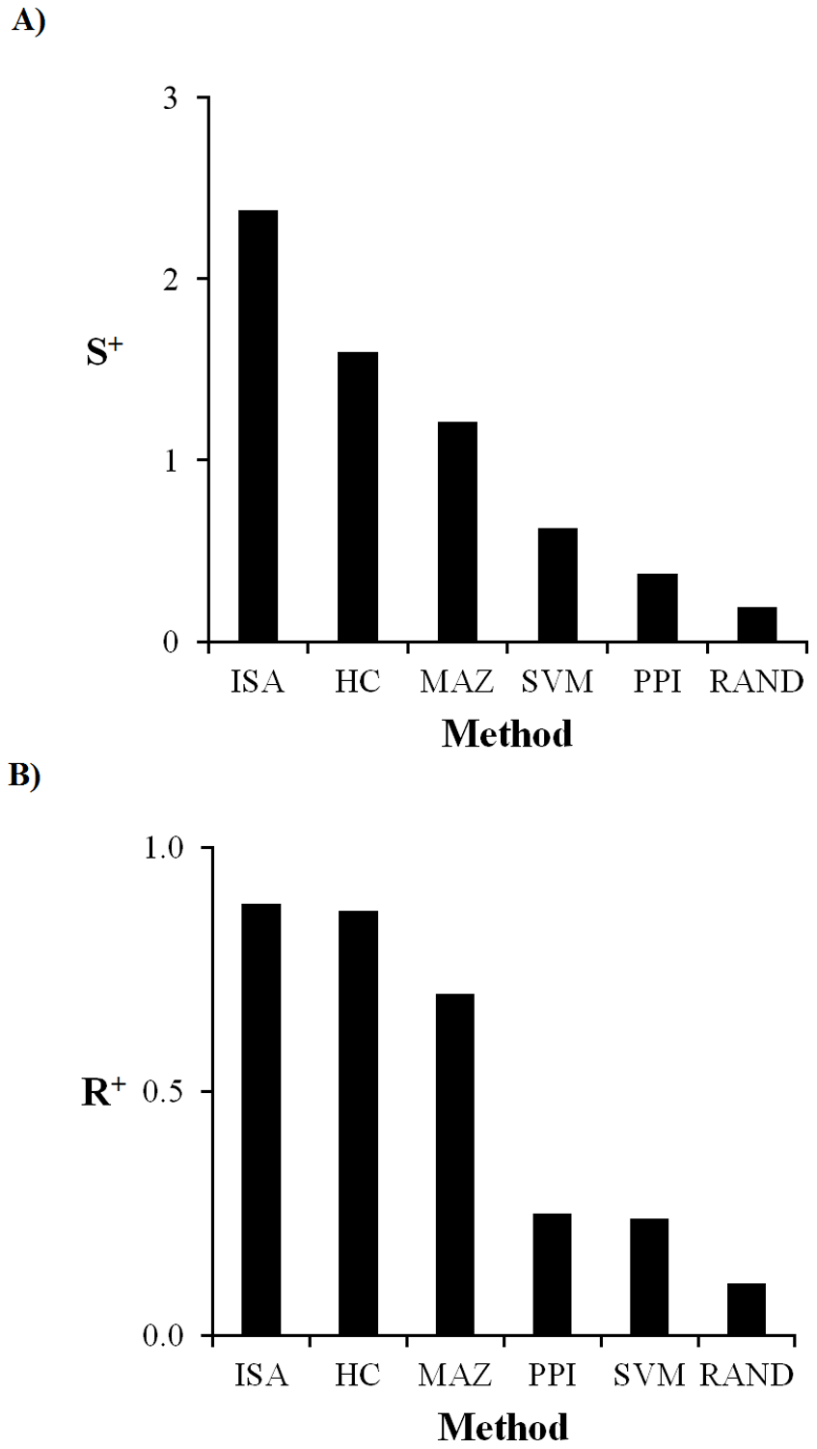
Module specificity and intra-module correlation. **A**) Tests for maximum module specificity, and **B**) maximum intra-module gene correlation. ISA, iterative signature algorithm gene co-expression modules; HC, hierarchical clustering gene sets; MAZ, gene sets composed of the top differentially expressed genes associated with injury indicator; SVM, support vector machines gene sets; PPI, protein-protein interaction network gene sets; RAND, gene sets composed of 100 genes selected at random. All method results were statistically significantly (*p*-value <0.05) different from the results generated using the random gene set.

We used the global specificity and correlation metrics defined in Equations (12) and (15) to evaluate the different methods' ability to generate gene sets for module construction. **Figure 2A** shows the global specificity and **Figure 2B** shows the global intra-set gene correlation computed for each of the investigated construction methods. In general, the ISA, hierarchical clustering, and maximum-fold-change-derived genes sets were better than the protein-interaction-derived and the SVM-derived gene sets for these metrics, with the random case showing the least specificity and correlation among the different groupings. Given that the ISA procedure produced the most coherent gene sets, we chose them for further analysis and characterization as gene *modules* associated with toxic response pathways. **Table S2** in the Supporting Information provides the gene membership of the ISA modules.

### Gene module mappings to injury indicators

We used Equation (8) to calculate module activation under conditions causing abnormal (+) instances of the 25 injury indicators, as well as those known to contain the nine chemical structure-activity classes listed in **Tables 1** and **2**. **Table S3** in the Supporting Information gives the calculated gene module map consisting of 78 rows of modules and 34 columns defined by injury indicator or structure activity classes. Each entry of this matrix corresponds to the module activation calculated using Equation (8).

**Figure 3:**
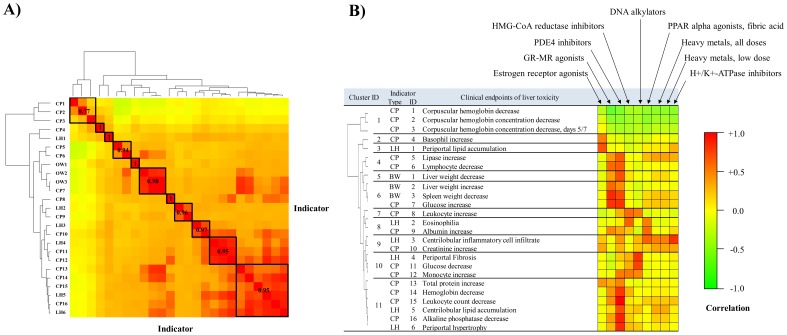
Clustering and analysis of injury indicators using module activation patterns. **A**) Correlation among injury indicators. The clinical endpoints used in **Table 1** are not independent, but highly correlated both from a biological point of view as well as from the gene transcription activation pattern. The hierarchical clustering dendrogram identifies the most related endpoints based on a Pearson correlation of iterative signature algorithm (ISA) module activation patterns. **B**) Correlation of injury indicators with structure activity classes. The clustering of the indicators is shown by a dendrogram at left; at center are the various injury indicators; at right is a heat map with elements equal to the Pearson correlation between the injury indicators at center, and the structure activity indicators arrayed across the top right. The Pearson correlation is determined using the covariance of the ISA module activation patterns of the injury indicators and structure activity classes. The Pearson correlation value in the first column of the table is the average intra-cluster correlation between indicators in the same cluster. CP, clinical pathology; LH, liver histopathology; OW, organ weight.

The ISA-constructed modules contain partly overlapping information, as the gene module-membership is not unique, in contrast to hierarchical clustering. The appearance of a gene in several different co-expression modules is consistent with the idea that similar molecular toxicity pathways can be activated under different conditions, and the idea that a gene can be part of more than one toxicity pathway. To account for the similarity of the modular response, we performed hierarchical clustering of the 25 injury indicators using 1 minus the Pearson correlation (1 – *r*) as the distance measure between indicators based on the module's activation patterns. **Figure 3A** shows the results of this clustering where we have defined 11 generalized indicator clusters based on similarity in module activation. We further categorized these groups in **Figure 3B** to link them to the injury indicator and to show their relationship to the different structure activity classes also present in the dataset.

The presence of several different classes of drugs and chemical toxicants in the data allowed us to match the adverse clinical diagnostic response (injury indicators) to these chemical classes. We used the correlation of module activation patterns between the chemical classes shown in **Table 2** and the injury indicators shown in **Table 1** to analyze these associations. **Figure 3B** shows the correlation pattern and highlights that drugs have multiple potential adverse effects in addition to their therapeutic effects [36]–[38].

For the cases where the exposure conditions that defined the chemical class activation patterns were not the same conditions/chemicals that caused the abnormal injury indications, we noted several adverse effect associations. *PDE4 inhibitors* and *Glucocorticoid-mineralocorticoid receptor (GR-MR) agonists* had the most wide-spread positive correlations with the injury indicators. Many PDE4 inhibitors are known to have a low therapeutic index and are associated with such side effects as nausea, vomiting, and weight loss [39]–[41]. Glucocorticoid receptor agonists are reported to have a diverse side effect profile, including weight gain, metabolic syndrome, lipodystrophy, hypertension, and fractures [37]. More specifically, glucocorticoids are reported to stimulate glucose production and decrease the number of circulating monocytes, eosinophils, and basophils [42]. These patterns of adverse effects were in qualitative agreement with our correlative analysis. Thus, the activity class *GR-MR agonists* had a positive correlation with glucose increase, lymphocyte decrease, leukocyte count decrease and negative correlation with glucose decrease, basophil increase, and monocyte increase in our analysis. Furthermore, estrogen receptor agonists are known to affect lipid profiles and metabolism [38], [43], [44], and we noted that the activity class *Estrogen receptor agonists* was most highly correlated with *Periportal lipid accumulation* (Pearson correlation, *r* = 0.85). The known association between DNA alkylators and liver fibrosis [45]–[47] was in agreement with the observed correlation between *DNA alkylators* and *Fibrosis* (*r* = 0.91). Thus, even though there was no overlap between the chemicals known to cause an adverse effect in this analysis, we were able to link the chemicals to their adverse effects based on the correlative analysis of the module activation patterns. These observations support the constructed co-expression modules as linkages to observable clinical injury associations.

**Figure 4:**
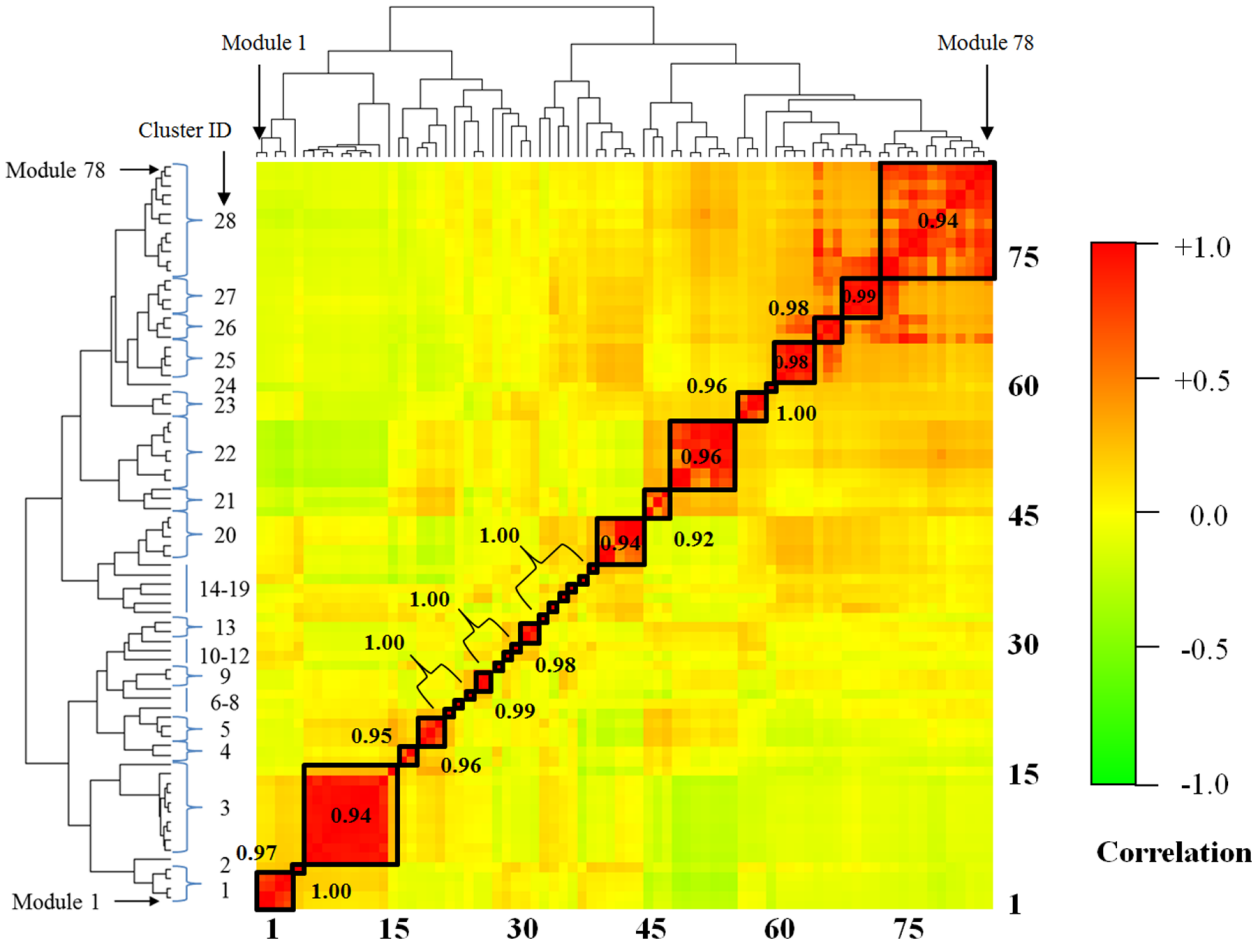
Clustering of the iterative signature algorithm (ISA) modules. By construction, the modules represent groups of genes co-expressed across a subset of the conditions, and they may share genes. The clustering gauges the independence of the modules and groups some modules together. A dendrogram of the clustering is shown at right, giving the module membership 1–78 of each of the 28 module clusters. The Pearson correlation is the average intra-cluster correlation between modules in the same cluster.

**Figure 5:**
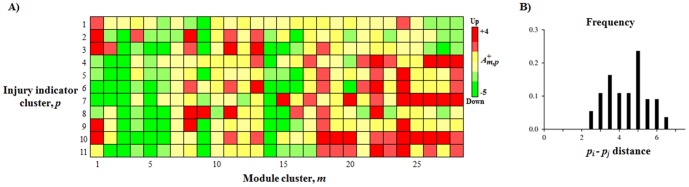
Activation pattern of module clusters. **5A**) Reduced representation of the each module cluster's activation patterns for the injury indicators shown in **Figures 3** and **4**. The illustration highlights the differences and similarities of each injury indicator based on their module activation patterns. **5B**) Shows the root-mean-square distance between all unique injury-indicator cluster pairs calculated using the averaged activation scores 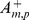.

Although the co-expression gene modules represent distinct but still partly overlapping molecular responses, we can also simplify the module characterization by clustering them into similar response modules. **Figure 4** shows the hierarchical clustering of the 78 modules using the similarity (Pearson correlations) of the activation pattern across the 25 injury indicators. The clustering reduced the number of co-expression modules into 28 clusters based on a minimum correlation cutoff of 0.90. We used the corresponding dendrogram to create the averaged activation patterns across the 11 reduced indicator clusters shown in **Figure 3A**. **Figure 5A** shows the corresponding reduced activation patterns calculated by averaging the Z-score over all indicators within an indicator cluster, and all modules within a module cluster. The Supporting Information **Table S4** provides the results before averaging over the module clusters. Each of the 11 reduced injury indicator clusters (except for hemoglobin levels) contained at least one module with an absolute 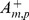 greater than 1.5, indicating that the constructed co-expression modules had a varied activation pattern that covered the bulk of the response inherent to the injury indicators. The **Figure 5B** shows the distribution of the root-mean-square activation distance between all unique injury-indicator cluster pairs. The minimum distance of 2.1 shows that the indicator clusters were well separated from each other and carried a characteristic activation pattern specific to the injury indicators.

### Linking injury indicator to KEGG pathways via module activation

We further analyzed the constructed gene co-expression modules by mapping them to KEGG pathways as outlined in the Method section. **Table 3** shows that the injury indicators listed on the right hand side are associated with distinct gene module patterns (center), and each of these gene module patterns is enriched with genes from a different set of KEGG pathways. For example, *Periportal lipid accumulation* was associated with up-regulation of genes in modules 1 and 27, which are enriched in genes involved with glutathione metabolism and the proteasome [48]. The up-regulation of proteasome pathways in the liver is consistent with proteasomal degradation of the regulatory binding protein (*Srepb1*), a transcription factor that activates lipid biosynthesis [49]. This shuts down lipid biosynthesis as a response to the high lipid levels associated with chemical injuries. Mechanistic links have also been recorded between *Eosinophilia* and fatty acid metabolism [50]–[52].

**Table 3:** KEGG pathway mapping.

Module cluster →	8	1	1	11	20	20	9	9	Injury indicator
KEGG pathway ↓ Module →	23	3	1	27	40	41	24	25	
Biosynthesis of unsaturated fatty acids	↑								Albumin increase & Eosinophilia
Butanoate metabolism	↑								
Fatty acid metabolism	↑								
Peroxisome	↑								
Propanoate metabolism	↑								
Valine, leucine and isoleucine degradation	↑								
Drug metabolism - CYP P450		↓							Spleen weight decrease
Metabolism of xenobiotics by CYP P450		↓							
Glutathione metabolism		↓							
Glutathione metabolism			↑						Periportal lipid accumulation
Proteasome				↑					
Natural killer cell mediated cytotoxicity					↑	↑			Periportal fibrosis
Regulation of actin cytoskeleton					↑	↑			
Leukocyte transendothelial migration					↑	↑			
Phagosome					↑	↑			
Pyruvate metabolism							↓		Alkaline phosphatase & Liver weight decrease
Pyruvate metabolism							↓	↓	Basophil increase

Pathways are at left, injury indicators are at right, and connecting gene co-expression modules are in the middle. Pathways are included only if they are associated with absolute module activation scores, 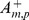, greater than 1.5, if they have Bonferroni-corrected *p*-values less than 0.05, and if there are at least six hits from the module in the pathway.

In our analysis, *Periportal fibrosis* was associated with up-regulation of modules 40 and 41 which are enriched in genes associated with activation of the phagosome, leukocyte transendothelial migration, regulation of the actin cytoskeleton, and natural killer cell-mediated cytotoxicity pathways [53]. These are all processes linked to fibrosis, e.g., when hepatocytes are injured, hepatic stellate cells migrate to the site of injury and transform into myofibroblasts, which produce large amounts of extracellular matrix proteins (ECM), such as collagen. Activated stellate cells produce cytokines and chemokines, which recruit and direct leukocytes to the injury site. Arriving leukocytes migrate through the endothelium to get to the injury site. Once at the injury site, leukocytes produce cytokines that cause the activated stellate cells to produce more collagen. A cycle occurs in which inflammatory (leukocytes) and fibrogenic (stellate) cells stimulate each other [53], causing production of ECM and ultimately scar tissue or fibrosis.

These observations showed that the different biochemical response pathways underlying different injury indicators could qualitatively be described by the difference in gene co-expression module activation patterns.

### Known biomarkers used in liver function tests

Injury-specific biomarkers found in the serum/plasma of both humans and rats have the potential to be used for diagnosing chemical toxicity and predicting adverse human health effects [19], [54]. Although a successful biomarker may be unrelated to co-expression or co-regulation of gene transcription, our module methodology attempts to map such processes through the concept of molecular toxicity pathways. Thus, to further characterize the co-expression modules, we examined their relationship to biomarker panels used in standard animal and human diagnostic tests for liver disease.

#### Co-expression modules linkage to ALT and AST

Alanine aminotransferase (ALT or *Gpt*) and aspartate aminotransferase (AST or *Got1*) are two clinically used serum biomarkers that have the potential to be generally informative of mammalian liver injuries through their serum levels [19], [54]. We analyzed their corresponding gene transcription in terms of individual gene activation (Z-scores associated with the log ratio of expression values) as well as activation patterns of modules containing these genes. Because genes in our construction may belong to multiple modules, we created an average activation score of those modules based on membership of the module clusters (**Figure 4**). Thus, the *Gpt* module activation pattern was constructed from averaging the 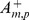-values across modules 43 and 44, whereas the *Got1* was created using modules 47–51.

**Figure 6:**
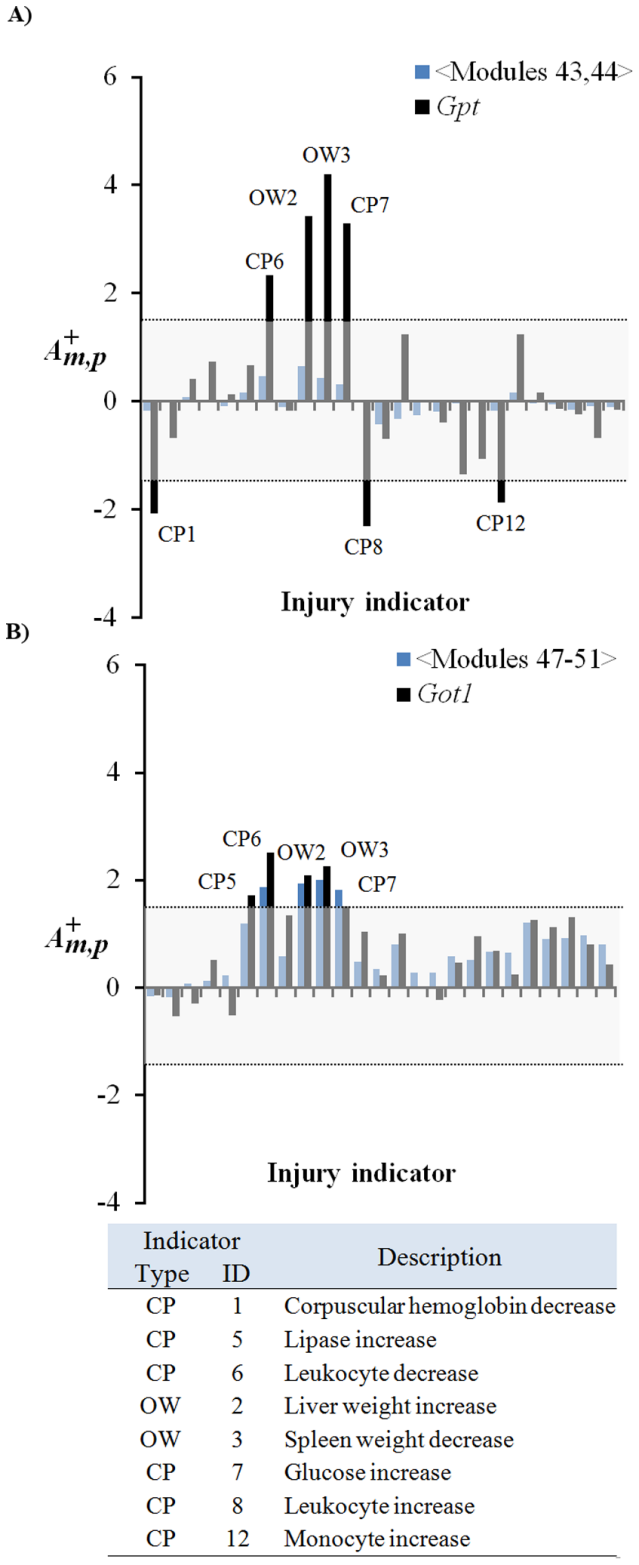
Activation patterns for selected modules and biomarker genes. Activation patterns shown correspond to the 25 injury indicators in **Table 1**. Labeled peaks represent average module activation score 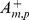 greater than 1.5 as calculated using Equation (8). **A**) The top graph shows averaged activation of modules 43 and 44 compared with the gene activation pattern of alanine aminotransferase (*Gpt*). **B**) The middle graph shows the average activation of modules 47 to 51 compared to the gene activation pattern of aspartate aminotransferase (*Got1*). CP, clinical pathology; OW, organ weight.

**Figure 6A** shows that the activation patterns of the modules containing *Gpt* were never significantly up- or down-regulated, whereas the pattern of *Gpt* expression was significantly (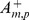 larger than 1.5) up- or down-regulated for seven injury indicators associated with abnormal clinical pathology endpoints. The gene activations relate *Gpt* to clinical pathology endpoints and body-organ weight changes, but not to any abnormal liver histopathology. This is consistent with the fact that ALT measurements, though highly informative at diagnosing liver injury, do not always correlate well with preclinical histopathology [54]. Importantly, the absence of module activation under conditions with a large increase in *Gpt* indicated a lack of a strongly co-expressed liver gene set that contained *Gpt*. **Figure 6B** shows that the activation patterns of the modules containing *Got1* were more similar to the individual gene activation pattern of *Got1* itself. Here, both activation patterns relate *Got1* to clinical pathology endpoints and body-organ weight changes, but again, not to any abnormal histopathology indications. With the exception of *Lipase increase*, both ALT and AST genes were indicative of increased activation of the same general clinical pathology and body-organ weight changes.

Our module construction emphasized the module activation pattern, not individual gene activation patterns, as central to the molecular toxicity pathways. This provides an avenue for detecting gene signatures and potential biomarker panels that can be associated conceptually with toxicity response pathways that are highly co-expressed. Given that the underlying data represents acute, non-fatal toxicity as captured via transcriptomics, our approach is limited. For example, it cannot identify chronic liver damage or injury due to non-specific deregulation, nor can it identify when proteins undergo enhanced excretion or leakage. While these processes may lead to robustly detectable biomarkers in biofluids, they are not necessarily informative of the full spectrum of possible liver injures.

#### Co-expression modules linked to the FibroSure [55] diagnostic test

**Figure 7:**
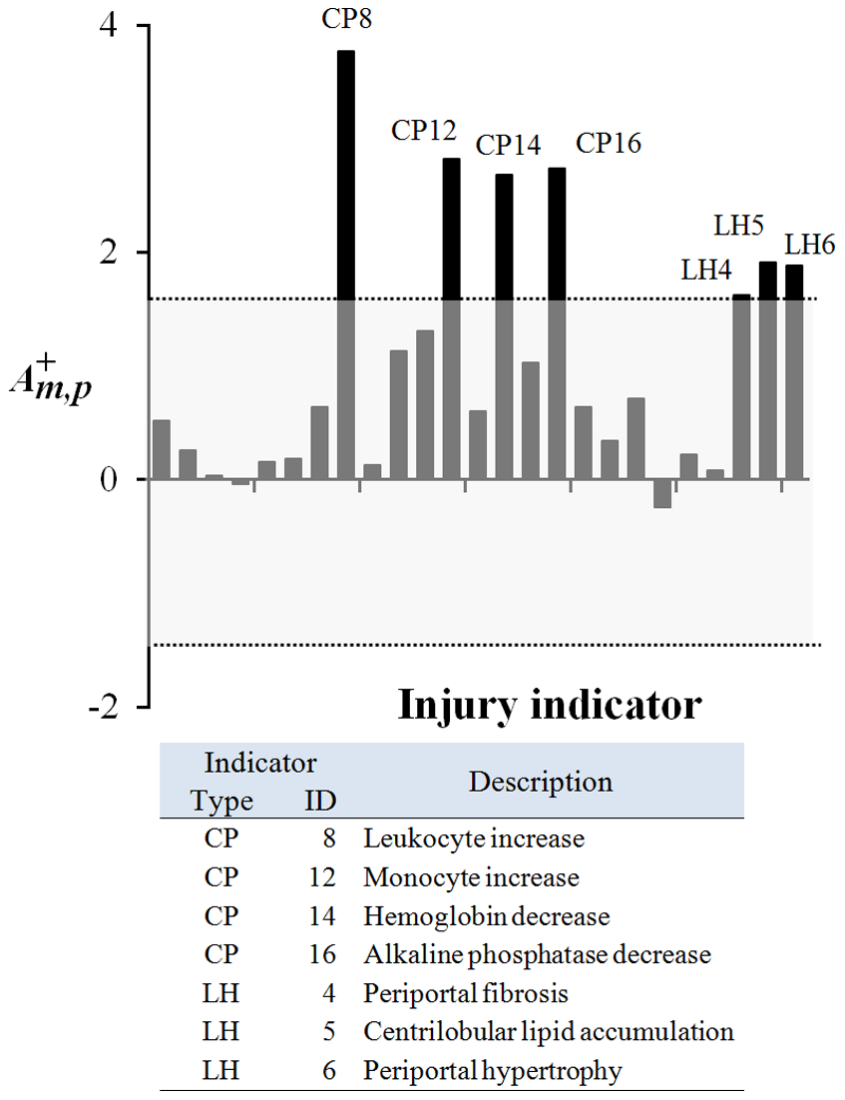
Module 55 activation across the 25 injury indicators. Activations shown represent the 25 injury indicators in **Table 1**. Labeled peaks represent a module 55 activation score 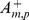 greater than 1.5 as calculated using Equation (8). CP, clinical pathology; LH, liver histopathology.

We further determined which of our 78 co-expression modules contained the gene markers used in FibroSure [55], a diagnostic test for human liver fibrosis, steatosis, and hepatitis. Out of the five proteins in the test, the gene encoding alpha-2-macroglobulin was present in module 55, whereas the alanine aminotransferase gene was found in modules 43 and 44. However, as discussed above, the latter two modules were not activated, as the activation score 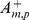 calculated using Equation (8) did not exceed the threshold of 1.5 for any of the injury indicators in **Table 2**. In contrast, **Figure 7** shows that module 55 was significantly (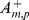 larger than 1.5) up-regulated for seven injury indicators associated with both abnormal liver histopathology and clinical pathology endpoints. Module 55 activation relates the FibroSure diagnostic endpoints of liver fibrosis and steatosis to the liver histopathology endpoint of *Periportal fibrosis* and *Centrilobular lipid accumulation*, respectively. Likewise, module 55 association with *Leukocyte increase* and *Monocyte increase* is consistent with the FibroSure diagnostic endpoint of hepatitis.

These observations showed how our computed gene co-expression modules could be linked, directly and indirectly, to clinical tests and established biomarkers for both animal and human liver injury. In order to explore the strengths of the co-expression modules and their capacity to describe liver injures, we next identified specific gene sets linked to select injuries.

### Gene sets as liver injury biomarker hypotheses

The construction of gene co-expression modules that broadly characterize chemical injuries to the liver can be used to select specific gene signatures that may be proposed as genes and proteins for future development of clinical biomarkers. We propose two general strategies that focused on either a specific module that is activated under chemical stress or a particular injury indicator.

#### Creation of liver injury gene signatures based on modules

**Figure 8:**
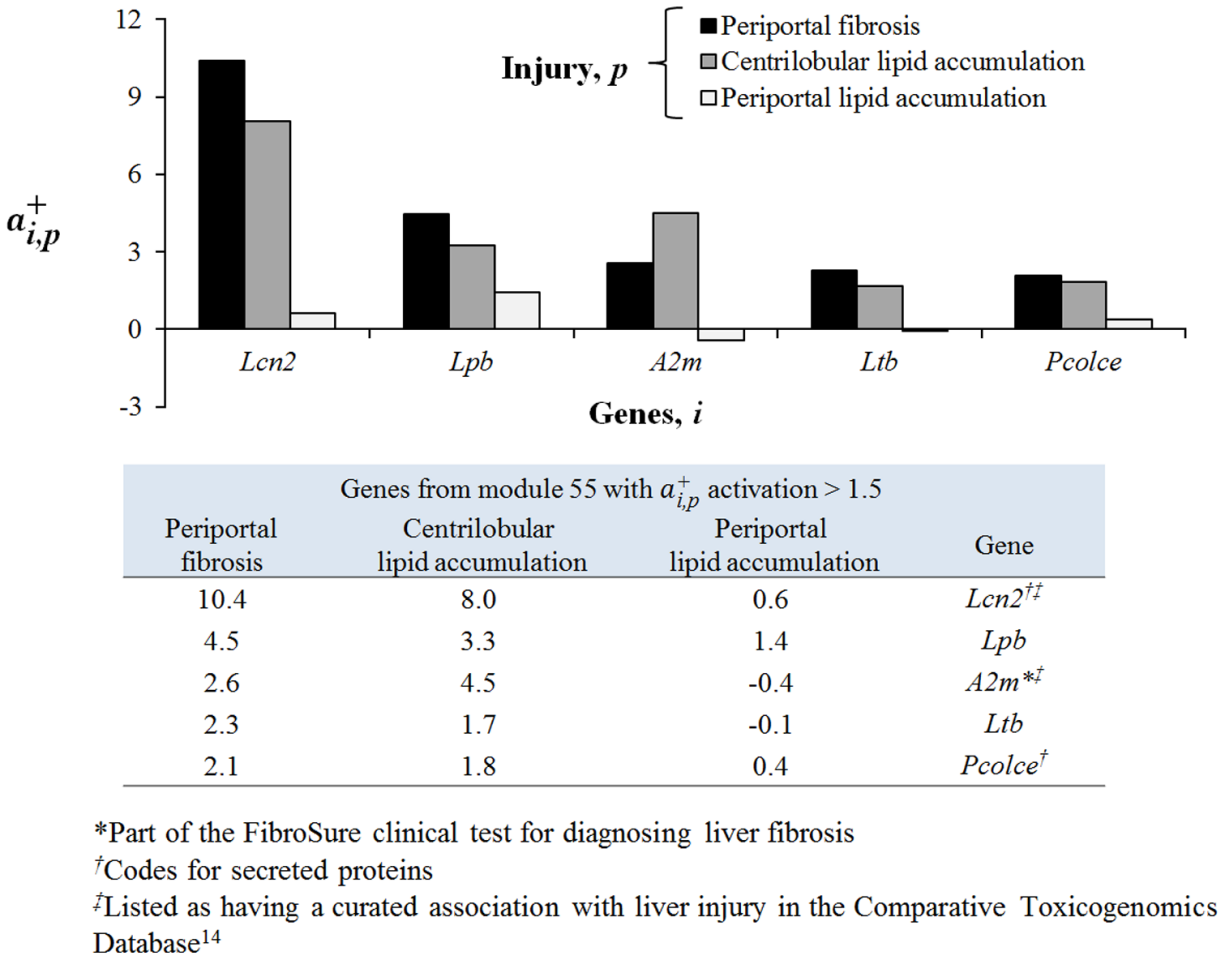
Activation of selected genes from Module 55. Selected genes show significant gene activation 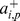 for fibrotic conditions. *Lcn2*, lipocalin 2; *Lbp*, lipopolysaccharide binding protein; *A2m*, alpha-2-macroglobulin; *Ltb*, lymphotoxin beta; *Pcolce*, procollagen C-endopeptidase.

Based on the association of FibroSure biomarker A2M with module 55, we examined all genes in this module with an activation score 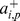 greater than 1.5 under conditions that can be linked to fibrotic injuries using Equation (16). **Figure 8** shows a bar plot of these genes and their activation levels for *Periportal fibrosis*, *Centrilobular lipid accumulation*, and *Periportal lipid accumulation*. As discussed above, *A2m* is up-regulated under fibrotic conditions, but both *Lcn2* (lipocalin 2) and *Lbp* (lipopolysaccharide binding protein), showed much larger magnitudes of activation under fibrotic conditions than did *A2m* itself. However, no gene activations could be significantly associated with *Periportal lipid accumulation*. Both *Lcn2* and *Pcolce* (procollagen C-endopeptidase enhancer) code for secreted proteins, and *Lcn2* has a known association with liver injury in the Comparative Toxicogenomics Database [20]. The genes listed in **Figure 8** thus constitute a plausible set of putative biomarkers of liver injury associated with fibrosis and centrilobular lipid accumulation.

#### Creation of gene signatures based on liver injury characteristics

**Figure 9:**
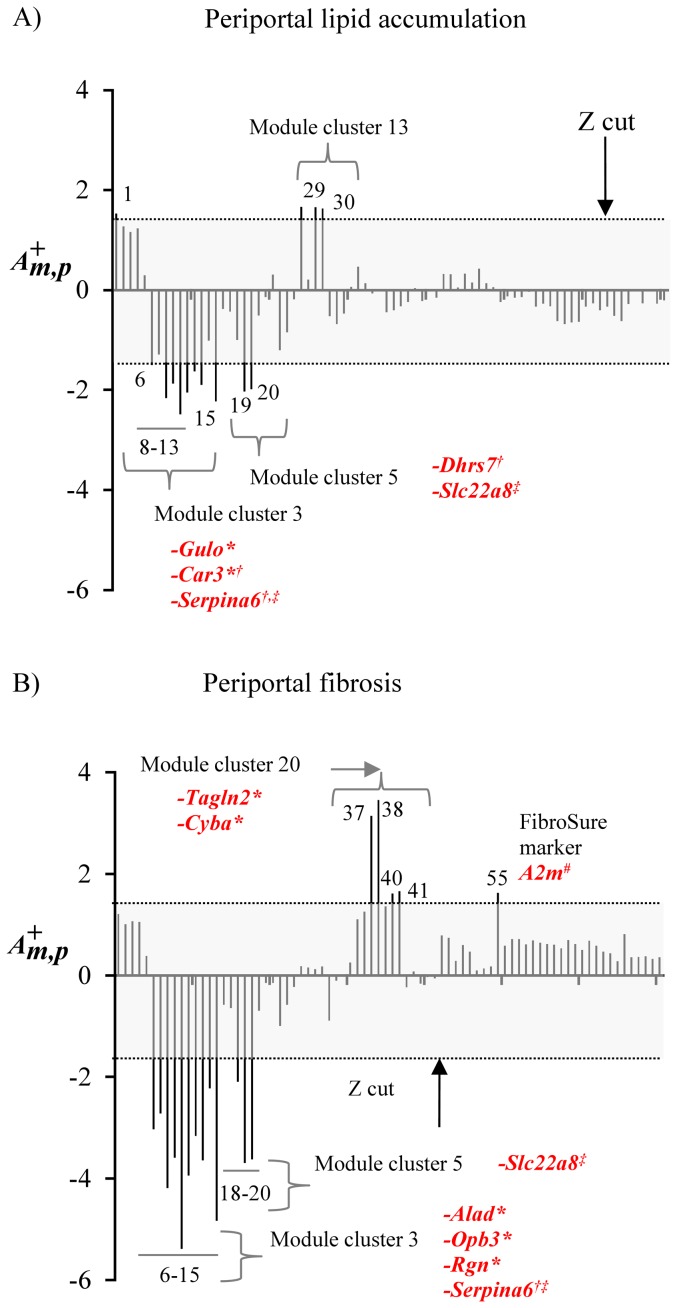
Module activation patterns for periportal lipid accumulation and periportal fibrosis. Module activation patterns for **A**) *Periportal lipid accumulation* and **B**) *Periportal fibrosis*. The grey box represents an absolute module activation score 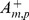 greater than 1.5 as calculated using Equation (8). Activation scores greater than the cut-off are labeled by their associated module numbers and module clusters. Modules are labeled with their center genes if the genes have a curated association with liver injury in the Comparative Toxicogenomics Database (*), if the genes code for secreted proteins (†), or if the genes are shared between periportal lipid accumulation and periportal fibrosis (‡). Modules are also labeled with member genes (not necessarily center genes) if they are part of the FibroSure biomarker set (#).

As a second example of signature selection, we simultaneously analyzed the activation profile of all 78 co-expression modules for two injury indicators, *Periportal lipid accumulation* and *Periportal fibrosis*. **Figure 9** shows the module activation 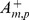 profiles for these two indicators as calculated using Equation (8). To find genes that were broadly characteristic of these modules we identified the center genes, as described in the Methods, derived from all modules that showed an activation of 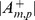 greater than 1.5. **Tables 4** and **5** list the characteristic genes for the two injury indicators, as well as functional gene annotations from the Rat Genome Database (RGD) [56].

**Table 4:** Gene signatures for *Periportal lipid accumulation*.

Module	Activation	Gene symbol	Gene name	Rat Genome Database terms[56]
30	↑	*Gpd1l*	Glycerol-3-phosphate dehydrogenase 1-like	Metabolic process; NADH metabolic process; negative regulation of peptidyl-serine phosphorylation
29	↑	*Cndp2*	CNDP dipeptidase 2 (metallopeptidase M20 family)	Proteolysis
27	↑	*Psma5*	Proteasome (prosome, macropain) subunit, alpha type 5	Ubiquitin-dependent protein catabolic process; ubiquitin/proteasome degradation pathway
1	↑	*Atpif1*	ATPase inhibitory factor 1	Erythrocyte differentiation; heme biosynthetic process; negative regulation of endothelial cell proliferation
19,15	↓	*Slc22a8*	Solute carrier family 22 (organic anion transporter), member 8	Glutathione transport; quaternary ammonium group transport; response to methotrexate
8-11	↓	*Serpina6*	Serine (or cysteine) peptidase inhibitor, clade A, member 6 (secreted)[57]	Glucocorticoid metabolic process
13	↓	*Gulo* *	Gulonolactone (L-) oxidase*	L-ascorbic acid biosynthetic process
6	↓	*Nrep*	Neuronal regeneration related protein	Axon regeneration; regulation of neuron differentiation; regulation of transforming growth factor beta receptor signaling pathway
20	↓	*Dhrs7*	Dehydrogenase/reductase (SDR family) member 7 (secreted)[57]	Encodes a protein that exhibits oxidoreductase activity
12	↓	*Car3* * [58]	Carbonic anhydrase 3 (secreted)[57]	Response to ethanol; response to oxidative stress

*Listed as having a curated association with liver injury in the Comparative Toxicogenomics Database [20].

**Table 5:** Gene signatures for *Periportal fibrosis*.

Module	Activation	Gene symbol	Gene name	Rat Genome Database terms[56]
37	↑	*Vim*	Vimentin	Aging; cellular response to fibroblast growth factor stimulus; decidualization
55	↑	*Cp*	Ceruloplasmin (secreted)[57]	Hypoxia inducible factor pathway; porphyrin and chlorophyll metabolic pathway
38	↑	*Tagln2* *	Transgelin 2	Muscle organ development
40	↑	*Unc93b1*	Unc-93 homolog B1 (*C. elegans*)	Antigen processing and presentation of exogenous peptide antigen via MHC class II; intracellular protein transport; toll-like receptor 3 signaling pathway
41	↑	*Cyba* *	Cytochrome b-245, alpha polypeptide	Cellular response to amino acid stimulus; cellular response to gamma radiation; cellular response to glucose stimulus
6	↓	*Alad* *	Aminolevulinate dehydratase	Cellular response to lead ion; heme biosynthetic process; response to activity; heme biosynthetic pathway; porphyrin and chlorophyll metabolic pathway
18-20	↓	*Slc22a8*	Solute carrier family 22 (organic anion transporter), member 8	Glutathione transport; quaternary ammonium group transport; response to methotrexate; bile acid transport pathway
9-11,13	↓	*Serpina6*	Serine (or cysteine) peptidase inhibitor, clade A, member 6 (secreted)[57]	Glucocorticoid metabolic process
15	↓	*Obp3* *	Alpha-2u globulin PGCL4 (secreted)[57]	Extracellular region
8	↓	*Rgn* *	Regucalcin	Cellular calcium ion homeostasis; positive regulation of ATPase activity; regulation of calcium-mediated signaling
7	↓	*Slc13a4*	Solute carrier family 13 (sodium/sulfate symporter), member 4	Sodium ion transport; transmembrane transport
14	↓	*Slc17a2*	Solute carrier family 17, member 2	Transmembrane transport
12	↓	*Ust5r*	Integral membrane transport protein UST5r	Integral to membrane

*Listed as having a curated association with liver injury in the Comparative Toxicogenomics Database [20].

In the case of periportal lipid accumulation (**Figure 9A**, **Table 4**), *Gulo* (module 13) and *Car3* (module 12), are associated with liver injury in the CTD [20], and *Serpina6* (a member of modules 8-11) and *Dhrs7* (a member of module 20) code for secreted proteins. In the case of periportal fibrosis (**Figure 9B**, **Table 5**), *Tagln2* (module 38), *Cyba* (module 41), *Alad* (module 6), *Opb3* (module 15), and *Rgn* (module 8) are associated with liver injury in the CTD [20], and *Opb3* and *serpina6* code for proteins that are secreted. Although part of these signature panels overlap (*Slc22a8* and *Serpina6* are common to both injury indicators), the fact that some of these genes are already known to be associated with liver injury suggests that these genes sets may be used to generate potential biomarker panels for chemically-induced liver fibrosis and steatosis.

#### Creation of a general liver injury gene signature

Finally, we analyzed the activation profile of all 78 co-expression modules for all injury indicators simultaneously. We created a general liver injury panel by collecting 69 center genes from modules with an activation of 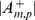 greater than 1.5 under conditions causing any of the injury indicator types. **Table S5** in the Supporting Information lists the general liver injury gene signature. Out of the 69 selected genes in **Table S5**, 11 (16%) are known to be associated with liver injuries in the CTD. **Table 6** shows genes associated with liver disease endpoints that include *1*) blood chemistry (anemia: low hemoglobin), *2*) fatty liver (accumulation of triglyceride droplets), *3*) fibrosis/cirrhosis (scar tissue formation), and *4*) necrosis (non-programmed cell death). Among these genes, *Sod2* was associated with multiple degrees of severe disease, while the others could potentially be used to stratify the injury severity. Both *Gulo* and *Car3* appear as markers of *Periportal lipid accumulation* in **Table 4**, and *Obp3* and *Rgn* as markers of *Periportal fibrosis* in **Table 5**. Thus, the identified genes provided a complex signature for a broad range of liver disease endpoints.

**Table 6:** Selected general liver injury signature genes with known disease annotations in the Comparative Toxicogenomics Database [20].

Disease/Pathology	Genes
Blood chemistry, anemia	*Sod2*
Fatty liver	*Sod2*
Fibrosis/cirrhosis	*Sod2, Hao2, Sult1e1, Got1, Gulo, Obp3, Bdh1*
Necrosis	*Sod2*
Liver neoplasms	*Sod2, Rgn, Anxa2, Car3, Gstp1*
Carcinoma	*Sod2, Gstp1*

### External validation

**Figure 10:**
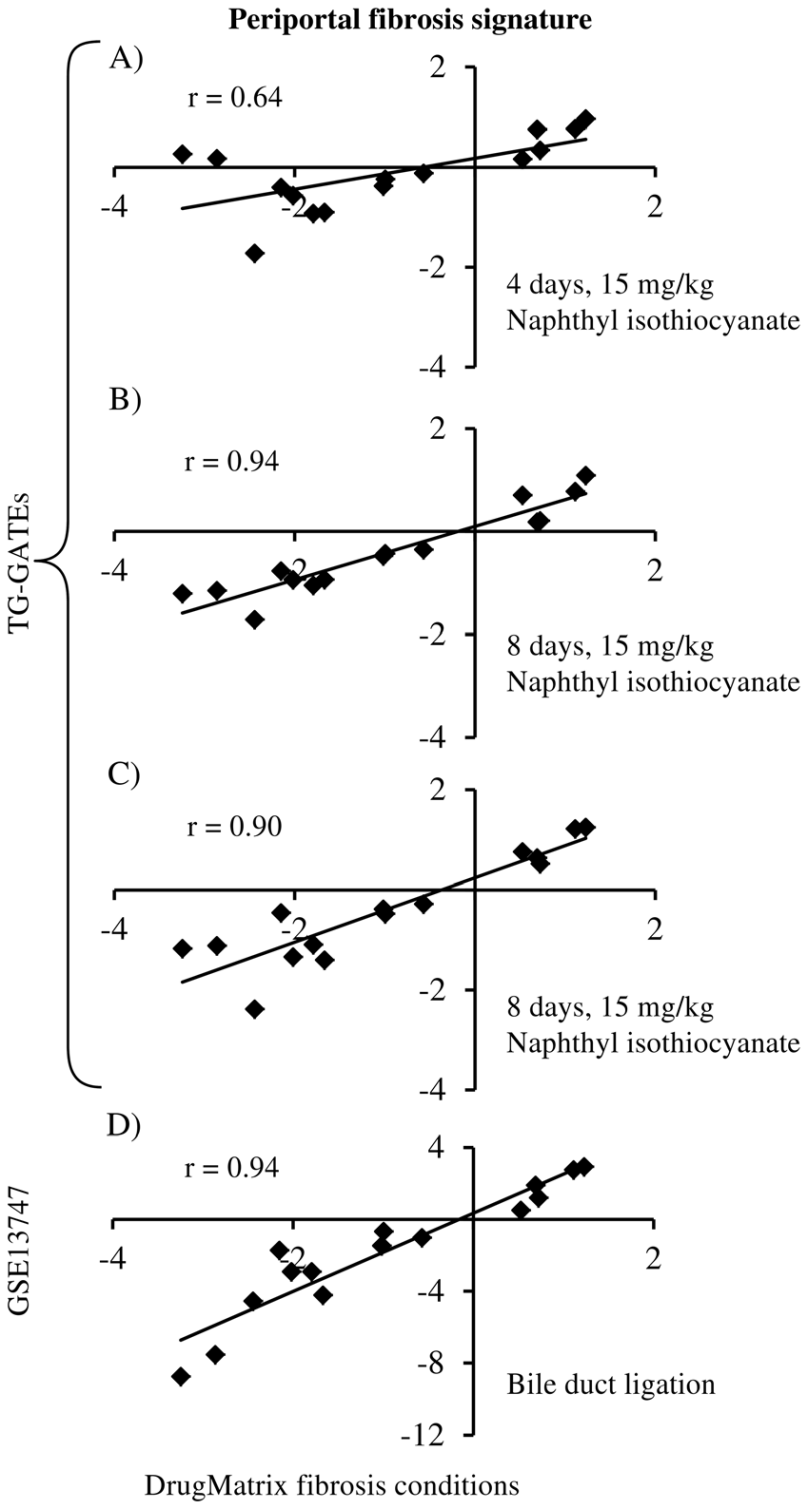
Validation of external datasets. Scatter plots show the correlation of the log-ratios between DrugMatrix data and external datasets for the periportal fibrosis gene signature. Comparison of the log-ratios in DrugMatrix periportal fibrosis conditions with **A**) 15 mg/kg of naphthyl isothiocyanate at four days of exposure obtained from the Toxicogenomics Project-Genome Assisted Toxicity Evaluation System (TG-GATEs), **B**) 15 mg/kg of naphthyl isothiocyanate at eight days of exposure obtained from TG-GATEs, **C**) 15 mg/kg of naphthyl isothiocyanate at 15 days of exposure obtained from TG-GATEs, and **D**) liver fibrosis produced by bile duct ligation obtained from GSE13747.

We further evaluated our gene signatures using external datasets collected from the TG-GATEs database and GEO. In the TG-GATEs database, high dose (15 mg/kg) of naphthyl isothiocyanate at four, eight, and 15 days exposures produced periportal liver fibrosis. For the genes in the periportal fibrosis gene signature, we compared the log-ratios in the DrugMatrix dataset to each of the three exposures and **Figure 10** **A–C** shows the observed correlation between these datasets. All the three exposure conditions exhibited positive correlation (*r*>0.6) with the DrugMatrix data. The four, eight, and 15 days exposures had correlation coefficient of 0.64, 0.94, and 0.90, respectively. Next, we evaluated the same fibrosis gene signature in a different dataset from GEO (GSE13747). In this dataset, liver fibrosis was induced by bile duct ligation. **Figure 10** **-D** shows the observed correlation between log-ratios of periportal fibrosis signature genes in DrugMatrix and GSE13747 dataset. Similar to the above results, we found the signature genes exhibit positive correlation (*r* = 0.94) in this dataset. These results show that genes that were identified to be relevant to liver fibrosis in our study behaved in a similar manner in external and independent fibrosis datasets.

**Figure 11:**
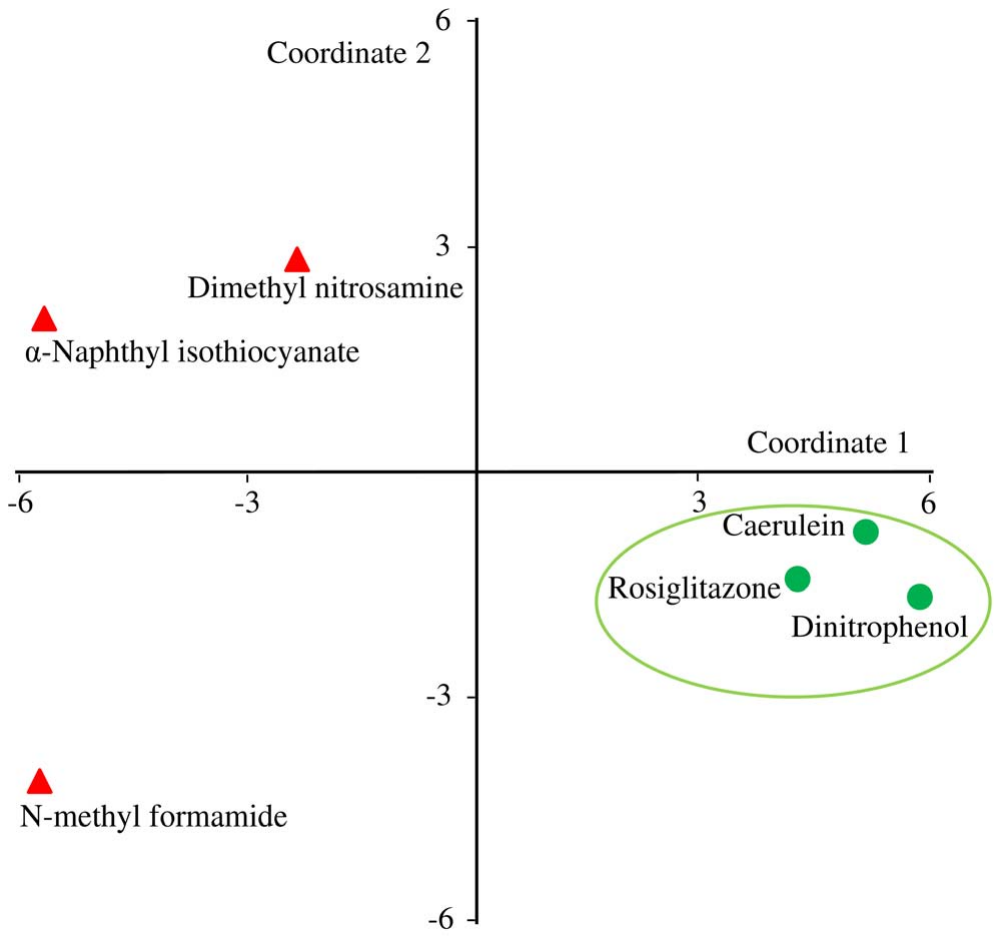
Analysis of exposures in GSE5509 using the general liver injury gene signature. Multidimensional scaling (MDS) plot of six chemical exposures in GSE5509 using the general liver injury gene signature. This figure shows the ability of the genes in the general liver injury signature to separate toxicants from non-toxicants. Rosiglitazone, caerulin, and di-nitro phenol, the non-toxic compounds in this set are marked in green circles. α-Naphthyl-isothiocyanate, dimethyl nitrosamine, and N-methyl formamide are the toxic compounds in this set, and they are marked with red triangles. In the MDS plot, the non-toxic compounds clustered separately form the toxic compounds. We have highlighted the non-toxic compounds within a green circle.

Finally, we evaluated the general liver injury gene signature using GEO dataset, GSE5509. In this dataset, gene expression data were collected from three toxic compounds (α-naphthyl-isothiocyanate, dimethyl nitrosamine, and N-methyl formamide) and three non-toxic compounds (rosiglitazone, caerulin, and di-nitrophenol). We used our general liver injury genes and evaluated the ability to group these two classes separately. **Figure 11** shows the MDS plot where we can see that the three non-toxic conditions grouped separately from the toxic conditions. These results provide an external validation and verification of our gene signatures.

## Conclusion

We have implemented a bioinformatics approach for selecting groups of co-expressed genes to classify different aspects of liver injury caused by drugs and chemical toxicants. From a range of different ways to construct such gene sets, we selected an iterative method (ISA) that produced gene modules based on sets of partly overlapping co-expressed genes. These modules were both descriptive of and specific to all the general pathology and liver histopathology assessments associated with the 200 chemicals administered at multiple sub-lethal doses and time points in male Sprague Dawley rats.

These modules consisted of genes that were highly co-expressed under the same set of exposure conditions and exhibited large activation changes under conditions causing abnormal injury indications. This provided modules that were both specific to an injury and contained genes that could be hypothesized to belong to a common biological process, inferred via the connection between co-expression and co-regulation. The common biological background of the toxic response processes were inferred from literature examples of specific genes in particular modules, and some of the identified genes encode proteins that are already part of clinically used diagnostics tests. As such, the modules may be useful for constructing gene signatures that could capture disease states and disease progression associated with chemical injuries. Focusing on injuries and health effects potentially allows us to capture the medically relevant aspect of chemical injuries, without resorting to large-scale *in vivo* characterizations of the multitude of potentially harmful chemicals we encounter in the environment.

We used the modules to examine different approaches to create genes set signatures derived from the entire dataset and based on module activation, fibrotic and steatotic injuries, or general liver injures. These genes sets were enriched with genes with known associations to known liver disease in the Comparative Toxicogenomics Database [20] and were descriptive of a broad range of clinical outcomes. Most of these signature gene sets currently have no direct associations with liver disease and, thus, provide a robust basis for developing predictive gene and protein biomarker panels for early diagnosis of toxic liver injuries.

The overall value of the computational approach was that we could readily integrate genome-scale amounts of biological data for a large number of different chemical exposure conditions with *in vivo* measurement of clinical chemistry and histopathological injury indications. In the presented module creation approach, we showed that it was computationally possible to find modules that were enriched in known liver-disease biomarkers, as well as being specific to particular liver injuries such as fibrosis. The disadvantage of the computational approach is that ultimately the conclusions drawn from the data rely on correlative and mathematical constructions that are not necessarily reflective of the underlying biological mechanisms. Correlative behavior is not necessarily related to causality; hence, even though the identified biomarker candidates can be proposed as strong hypotheses, they must still be experimentally verified in independent studies.

## Supporting Information

Table S1 **The resultant log-ratio Z-score matrix containing 7, 826 genes by 640 conditions.**
(XLSX)

Table S2 **ISA determined gene co-expression modules and their gene membership.**
(XLSX)

Table S3 **Gene module map containing 78 rows of modules and 34 columns defined by injury indicator or structure activity classes where each entry of the matrix corresponds to the module activation.**
(XLSX)

Table S4 **Module cluster activation patterns before averaging over the module clusters.**
(XLSX)

Table S5 **General liver injury gene signature set.**
(XLSX)

Script S1 **ISA module generation script.** R script and input files used to generate ISA modules in this work.
(7Z) Click here for additional data file.
